# Microwave-Assisted Production and Regeneration of Granular Expanded Graphite for the Adsorption of Cationic Dye Methylene Blue from Aqueous Solutions

**DOI:** 10.3390/ma19132884

**Published:** 2026-07-06

**Authors:** Alireza Faraji, Donatella Caniani, Delfina Bersano, Salvatore Masi, Ignazio M. Mancini, Linda Y. Tseng, Tanju Karanfil

**Affiliations:** 1 Department of Engineering, University of Basilicata, Viale dell’Ateneo Lucano, 10, 85100 Potenza, Italy; afaraji@g.clemson.edu (A.F.); salvatore.masi@unibas.it (S.M.); 2 Department of Environmental Engineering and Earth Sciences, Clemson University, 230 Kappa Street, Clemson, SC 29634, USA; 3 Department of Health Sciences, University of Basilicata, Viale dell’Ateneo Lucano, 10, 85100 Potenza, Italy; donatella.caniani@unibas.it (D.C.); ignazio.mancini@unibas.it (I.M.M.); 4 Eni S.p.A.—Natural Resources, Wind and Marine Energy Research Center, Via Felice Maritano, 26, 20097 San Donato Milanese, Italy; delfina.bersano@eni.com; 5 Civil Engineering Department, The City College of New York, 160 Convent Avenue, New York, NY 10031, USA

**Keywords:** adsorption, methylene blue dye, microwave irradiation, response surface methodology, thermally expanded graphite

## Abstract

**Highlights:**

**What are the main findings?**

- Microwave was used to improve expanded graphite as an adsorbent.
- The resulting adsorbent can be regenerated with microwave minimal weight loss.
- The created adsorbent was able to remove 94% methylene blue, a cationic dye, from freshwater.

**What are the implications of the main findings?**

- Microwave is an energy-efficient method in different environmental pollution applications.
- Applying expanded graphite with microwave and granularization improved its regenerability.
- The adsorbent in this study has the potential to remove dyes from different water matrices.

**Abstract:**

This study investigated the use of microwave (MW) to enhance the performance of a granular thermo-plasma expanded graphite (GTPEG) to remove an organic cationic dye, methylene blue (MB), from freshwater, taking the benefits of MW-assisted regeneration. Expanded graphite (EG), low-density and mesoporous, has been proposed previously as a means of extracting organic compounds from water due to its hydrophobicity and weak polarity. We granularized EG to facilitate its subsequent regeneration and reuse and separation, and MW was used to improve the adsorption of EG. The most important factors affecting adsorption were determined using response surface methodology, along with 3-D graphics illustrating the relationship between several variables. The experimental results show that 94% of MB adsorbed onto MW-GTPEG in deionized distilled water within 20 min. Additionally, adsorbent regeneration using MW did not affect the adsorption capacity and rate after 10 cycles albeit with a small weight loss of <8%. From our results, we believe that the main factor affecting the adsorption was the physical characteristics of the adsorbent, with other minor factors such as the adsorbent dose and initial concentration. Our findings suggest that MW irradiation may be a promising alternative for enhancing and regenerating GTPEG.

## 1. Introduction

Organic pollution from textiles, paper, and printing factories has a negative impact on both human and aquatic life. Due to their toxic nature, these organic pollutants lead to high chemical oxygen demand (COD), biological oxygen demand (BOD), decreased light penetration into surface water, and disruption in the development of microorganisms in aquatic life [1]. The US Environmental Protection Agency (EPA) is improving the safety of water resources and protecting human health by identifying hazardous waste from the dye and pigment industry. Wastes from specific dyes, pigments, food, drugs, and cosmetic colorants (FD&C) are recorded as EPA hazardous waste K181. By law, these wastes are managed under the Resources Conservation and Recovery Act (RCRA), over which EPA has authority and focuses on the total quantity of chemical components of concern in waste with the biggest risk [2]. Specifically, dyes are a prevalent synthetic contaminant found in aquatic environments due to their extensive use and consequential environmental implications. Their resistance to photodegradation leads to substantial challenges in terms of their removal from water. Methylene blue (MB), a well-known cationic and primary thiazine dye with a molecular formula of C_16_H_18_N_3_ClS, was employed here in this study because of its considerable aqueous solubility and potential risks to river and surface water ecosystems.

There are generally three main methodologies for the removal of dyes, including biological, chemical, and physical techniques. Biological methodologies involve cultivating organisms or harvesting the catalysts they produce, including but not limited to algae, enzymes, fungi, or bacteria. Electrochemical degradation, Fenton reaction, oxidation, ozone treatment, photochemical degradation, and UV irradiation are all chemical processes often employed for the degradation of dyes. Various physical approaches, including adsorption, coagulation or flocculation, ion exchange, membrane filtration, nanofiltration or ultrafiltration, and reverse osmosis, have been employed for the purpose of dye removal by mass transfer [3]. Table S4 presents a comparative analysis of the effectiveness of commonly employed methodologies. Among them, adsorption is widely recognized as an ecologically sustainable process due to its low production of by-products, excellent efficiency, and cost-effectiveness in reducing dye pollutants. Activated carbon (AC) is attracting considerable interest as an adsorbent due to its favorable characteristics, including a large specific surface area and a very rapid adsorption rate. Nevertheless, the higher cost associated with AC applications can inhibit their use in certain applications [4]. On the other hand, different types of low-cost adsorbents were applied for various heavy metals and synthetic dyes. And among them, snail and crab shells [5], a bagasse-bentonite mixture [6,7], algae waste-bentonite [8], leaves [9], silica-synthesized adsorbents [10], AC from different precursors [11], and Bali cow bones hydrochar powder [12] were reported as efficient and facile processes for the removal of various heavy metals (Cu, Pb, Cd, Zn, Ni) and synthesized dyes (methylene blue, methyl red, Congo red, etc.).

The production of expanded graphite (EG) has been extensively researched; however, little information exists in the literature regarding the regeneration of EG. Expended graphite is a low-density (0.002–0.010 g cm^−3^) and mesoporous carbon-based material [13]. It can adsorb organic compounds selectively owing to its hydrophobic nature and weak polarity [14]. EG’s structure includes numerous layers and interlayer space. The method for its production is, firstly, mixing natural graphite with nitric acid (HNO_3_) and sulfuric acid (H_2_SO_4_). After the graphite reacts with acids, the filtered mixture is washed with water and dried in an oven. The resulting material is ready to be expanded in a furnace at a temperature of 900 °C [15]. EG has been used in removing diesel oil [16,17,18,19,20], biomedical waste [21], heavy metals [22,23], and organic dyes [13,24]. Moreover, EG is recyclable and can be conveniently disposed of, if required [25,26]. The potential of EG in dye removal from aqueous solutions has been studied by Li et al. (2008) [27]; however, this study showed that EG demands a long time to adsorb target pollutants. In another study, Zhao and Liu (2009) used modified expanded graphite (MEG) [28]; their results of MB removal show that a basic pH, a higher initial dye concentration, and a high temperature could increase the adsorption performance. Nevertheless, the potential for regeneration of MEG was not addressed. Hoang et al. (2019) used microwave (MW) irradiation to synthesize EG [1]. The graphite intercalation compound (GIC) [1], which was subjected to MW irradiation, resulting in the production of EG with measured surface area of EG around 30 m^2^ g^−1^. The researchers employed ethanol as a solvent for MB desorption, thus regenerating the material, and they found that this method of regeneration led to a notable decrease in adsorption efficiency [1] and to being time- and energy-consuming. In their study, Wu et al. (2021) employed an expanded graphite/Fe_3_O_4_ composite that was synthesized by an explosive combustion technique utilizing black powder (a mixture of potassium nitrate, sulfur, and charcoal), and the authors emphasized the absence of harmful waste elements resulting from this methodology; however, the discussion of regeneration experiments related to dye removal was also omitted [29].

Microwave irradiation for the production and regeneration of EG also has not been explored extensively. Microwave irradiation has been demonstrated to be an efficient technology for thermal regeneration due to its quick heating rate, high heat transfer efficiency, and short contact times [30]. Microwave irradiation in granular form production can help modify the final material and enhance the adsorption process. The reason for the use of MW irradiation is that it can help remove impurities from pores and increase the macro-porosity, which acts as a connection particularly between the mesopores [31]. Furthermore, granular forms not only boost the adsorption process but also ease the regeneration of materials because of their shape when compared to powder forms. Several studies used chemical reagents, such as desorption with ethanol [1], 1% hydrochloric acid (HCl), H_2_SO_4_, and sodium hydroxide (NaOH) [32], as regeneration methods. Also, managing the solvents after regeneration is a major issue in chemical regeneration [33]. In contrast, thermal regeneration can be a possible alternative because it provides an easier process. However, the thermal regeneration process still suffers from considerable material weight loss due to friction, as well as burn-off and washout, resulting in a drop in adsorption capacity [30]. Additionally, energy consumption is another major concern in thermal regeneration. The conventional method (i.e., using an oven) demands preheating and, as previously mentioned, lacks practical and theoretical applicability. A recent study regenerated AC saturated with per- and polyfluoroalkyl substances (PFAS) by MW irradiation [30], and their results after five cycles show moderate weight loss and ~65% regeneration efficiency, showing the potential application of MW irradiation in the regeneration of AC materials.

Another issue in working with EG is its low density. To overcome this, granulation and impregnation of EG powder by calcium chloride (CaCl_2_) and sodium alginate can be viable options in a realistic view, since granular forms are easily separated from the solution and do not need further operation, such as centrifugation. This is something we included in our EG production in our study.

To reduce the number of possible experiments required in our study, we utilized response surface methodology (RSM) [34]. Response surface methodology is a set of statistical and mathematical methods used to improve processes by identifying the impacts of several factors. It is often used in chemometrics and was first used to describe the graphical view that resulted once the mathematical model was found to be fit. In the context of experimental design, RSM refers to a collection of mathematical and statistical methods based on the empiricism of fitting empirical models to experimental data. Traditionally, parameters influencing adsorption are determined through experiments by changing one factor at a time while keeping the other the variables the same. This optimization method, called “one variable at a time”, does not consider the interactions of different parameters; RSM considers all the combinations of runs and consumes more time and materials [35]. In contrast, multivariate statistical methods such as RSM are practical in adsorption optimization: it robustly plans experiments and setting models by analyzing the impact of simultaneous factor changes, aims to obtain the optimal experimental conditions in a shorter time and with fewer runs [36,37], and defines the behavior of a dataset by fitting a polynomial equation to create statistical predictions while simultaneously optimizing several parameters to obtain the best testing performance [38]. A wide range of literature presents and discusses the RSM method and its application in chemistry [39,40,41,42].

This study aimed to first modify the application of granular thermo-plasma expanded graphite (GTPEG) to acquire an adsorbent and investigate its performance in removing MB from aqueous solutions. To avoid the problems of separating the low-density EG from the solution, we impregnated EG with CaCl_2_ and sodium alginate. In addition, MW irradiation was used to remove impurities from pores as the second step in improving adsorption properties. Our hypothesis is that the GTPEG produced with MW removes MB with equal or better efficiency compared to other GTPEG. The second objective of this study was to evaluate low-energy regeneration technology in dye removal by performing adsorbent regeneration using MW. This study is novel because we used MW-assisted production of expanded graphite granules for dye removal while taking regeneration benefits, such as economical recycling in larger dye removal applications, into account.

## 2. Materials and Methods

### 2.1. Materials

Commercial thermo-plasma EG (TPEG) was supplied by Innograf (Potenza, Italy). Concentrated HCl and NaOH were used to adjust the pH values. Methylene blue (CI 52,015; chemical formula: C_16_H_18_CN_3_S; molecular weight: 319.86; maximum wavelength: 666 nm) was supplied by Carlo Erba Reagents as employed in this research. Also, sodium alginate and CaCl_2_ from Carlo Erba Reagents were used to prepare granular TPEG (GTPEG).

### 2.2. Preparation of GTPEG

**Figure 1:**
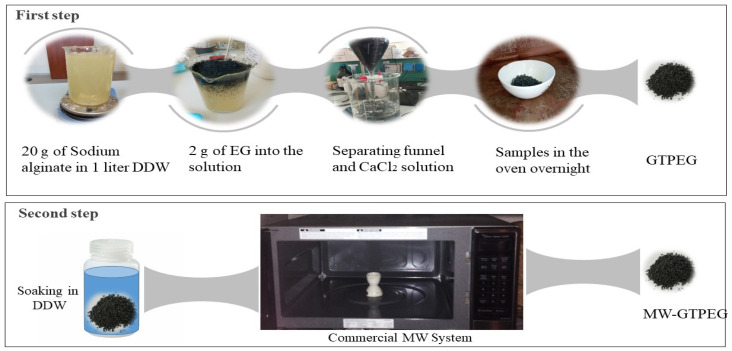
The stages of GTPEG preparation.

Thermo-plasma expanded graphite was used as the base material for the GTPEG production, as described in [43]. First, 20 g of sodium alginate was dissolved in 1 L of deionized distilled water (DDW) under mild magnetic stirring for two hours at room temperature. Two grams of TPEG were added to the solution under magnetic stirring until a transparent solution was obtained. Then, the solution was transferred into a separating funnel and transferred into a CaCl_2_ (2% in DDW) solution. The resulting granular material was then separated from the solution using a sieve. Finally, the material was put into an oven overnight at 105 °C. Consequently, the material obtained was named GTPEG. Figure 1 illustrates the process of GTPEG preparation in step 1. In the second step, also illustrated in Figure 1, a commercial MW source was used to remove impurities from the GTPEG. Two grams of GTPEG were soaked in a vial containing 50 mL of DDW. Then, after one hour of shaking the vial in an orbital shaker, saturated GTPEG was filtered to remove DDW. The moisture content of the material was determined to be approximately 100% before MW treatment. DDW stocked in expanded graphite pores allows the MW to heat up and paves the way for removing impurities that block the channels between pores within the mesoporous scale. In MW irradiation, the temperature gradient can be formed volumetrically throughout the volume of the material rather than through external energy such as convection (which is the case in conventional heating) [31]. DDW-saturated GTPEG was irradiated by the MW at different powers for different durations, and we determined that applying 510 watts for 2 min was enough to remove DDW from pores and purify the GTPEG’s structure (more details in Text S2). The final material was labeled microwave-GTPEG (MW-GTPEG) and kept dry for further utilization.

### 2.3. Batch Adsorption Experiments

The batch adsorption tests were carried out to determine the best conditions for the MB removal by adding different MW-GTPEG dosages (0.9, 1.8, and 3.6 g L^−1^) in a 25 mL vial of known MB concentrations (25 to 100 mg L^−1^). The experiments were performed at pH values between 3.6 and 11, obtained by adding 0.1 N HCl and 0.1 N NaOH. At a room temperature of 25 ± 1 °C, the vials were shaken with an orbital shaker at a constant speed of 200 rpm for various contact times (10, 20, and 30 min). Adsorption efficiency (R: %) and MB adsorption uptake of MW-GTPEG (q: mg g^−1^) were calculated using

(1) $$R=\frac{C_{i}-C_{e}}{C_{i}}\times 100$$

and

(2) $$q=\frac{V\times {(C}_{i}-C_{e})}{M},$$

respectively [44,45], where C_i_ and C_e_ (mg L^−1^) are the initial and the equilibrium concentrations, V (L) is the volume of the solution, and M (g) is the mass of the adsorbent.

### 2.4. Analysis and Characterization of Materials

Fourier transform infrared (FTIR) spectroscopy (Nicolet 670-912A0750, Thermo Scientific, Madison, WI, USA) was employed to determine the functional groups of TPEG and MW-GTPEG. A CHNS analyzer (carbon, hydrogen, nitrogen, and sulfur analyzer; Thermo Fisher Flash Smart, Waltham, MA, USA) measured the percentage of carbon, hydrogen, nitrogen, and sulfur. Ash contents were calculated by putting 100 mg of materials into a muffle furnace (SB80PC, Precision Scientific Group, GCA Corporation, Chicago, IL, USA) at 650 °C for 16 h. The surface area and pore distribution analysis were conducted by ASAP 2020 surface and porosity analyzer (Micromeritics, Norcross, GA, USA); samples were degassed at 90 °C for 12 h prior to the analysis. The BET theory is the most prevalent technique used to determine specific surface area. In the surface and porosity analyzer, samples are typically degassed by heating and pouring gas over the sample to eliminate the impurities. Then the sample is cooled with liquid nitrogen and analyzed by calculating the volume of N_2_ adsorbed at specific pressures. The pH of the point of zero charge (pHpzc) was measured by adding 20 mL of a 0.1 M sodium chloride (NaCl) solution into a beaker containing 100 mg of material, followed by shaking at 200 rpm for three days, and then measuring pH with a pH probe (VWR Symphony, Singapore). The concentration of MB was determined using about 3.5 mL sample volume with a UV-visible spectrophotometer (VACN 004 559 540, Varian, Australia) at the wavelength of 666 nm, which corresponds to the maximum absorbance of MB dye at neutral pH in a quartz cuvette with a 1-cm optical path. The calibrated curve was derived by plotting the recorded absorbances from the spectrophotometer versus different MB concentrations, with a coefficient of correlation (R^2^) of 0.9995.

### 2.5. Process Variables (Response Surface Methodology, RSM)

This study employed a flexible optional design structure to accommodate a custom model among the various matrix designs using the software Design-Expert 13 by Stat Ease^®^ with the response surface tool under standard designs and the optimal (custom) option (statease.com). In this study, linear or square polynomial functions were used to characterize our system, through modeling and displacement of experimental circumstances, until optimized [46]. It considered a more comprehensive range of categorical factors with 23 batch adsorption tests to optimize the R (Equation (1)) and q (Equation (2)) of MB adsorption onto MW-GTPEG. The four independent variables, including contact time (min), initial concentration of MB solution (mg L^−1^), adsorbent dose (g L^−1^), and pH of MB solution, were investigated for their effects on adsorption. This selection was based on previous lab reports, literature on dye adsorption, and the initial studies carried out in the laboratory [1,36] (in addition to the list in Table 10). It is worth mentioning that all batch tests were performed at room temperature (25 °C). The range of parameters optimized in this research is presented in Table 1. The material dose was calculated using TPEG powder; although sodium alginate and CaCl_2_ might have also contributed to adsorption, we did not investigate their adsorption capacity in our study.

### 2.6. Adsorption Isotherms, Adsorption Kinetics, and Thermodynamics of Adsorption

Experimental data were fit to Langmuir, Freundlich, Temkin, and Dubinin–Radushkevich [48] models to understand the adsorption process at various MB concentrations (5 to 100 ppm) for a 20 min (time needed for adsorption equilibrium > 90% adsorption rate) shaking time at 200 rpm (conducted for all experiments in this study), without modification of pH (5.7 close to the real condition). Table S5 provides equations and parameters for these models.

For the kinetics studies, 1.8 (g L^−1^) of adsorbent was added to a beaker containing 150 mL of 100 (mg L^−1^) of MB and stirred at 200 rpm with a pH of 5.7 at 25 ± 1 °C. At specific time steps (0 to 60 min), 1 mL of the solution was removed to measure the concentration. Then the uptake was determined for each time step. The experimental data were fitted to a pseudo-first order and a pseudo-second-order mathematical model (Table S6).

Assessing the experiments’ thermodynamics can help examine the interactions between spontaneity, free energy, and temperature during the process. To evaluate the thermodynamics of our experimental system, batch experiments were tested at various temperatures (i.e., 293, 299, and 311 K) in a shaking bath. The experimental conditions included 1.8 g L^−1^ of MW-GTPEG, 25 mL of MB solution (100 mg L^−1^), pH 5.7, and 200 rpm shaking speed for 20 min. The experimental parameters were based on the RSM results in Table S3. The relevant thermodynamic equations are listed in Table S7.

### 2.7. Regeneration and Reusing of MW-GTPEG

Regeneration studies were also performed to demonstrate the sustainability of materials and possible scale-up opportunities. Therefore, MW-GTPEG exhausted by MB were irradiated by a commercial MW oven (Panasonic, more details in Table S8); MW-assisted regeneration was used because of its lower energy consumption, fast and efficient implementation in MW absorbing materials like activated carbons [49], and effectiveness in regenerating activated carbons. For this purpose, adsorbents were regenerated for 10 cycles. Power and irradiation time are the principal factors in MW treatment. However, lower power values (500–700 watts) needed longer (8–10 min) to remove the MB from the adsorbent. Therefore, based on preliminary experiments, 1100 watts and 5 min were picked for this study to desorb MB from MW-GTPEG pores.

## 3. Results & Discussion

### 3.1. Characterization of Materials

MW-GTPEG and EG were characterized by determining the functional groups, surface area analysis (surface area and porosity via BET adsorption), carbon, hydrogen, nitrogen, and sulfur contents, ash contents, and pHpzc (pH point of zero charges) to understand their physical and chemical characteristics. The SEM analysis of EG materials was reported in our previous publication [43].

#### 3.1.1. FTIR

**Figure 2:**
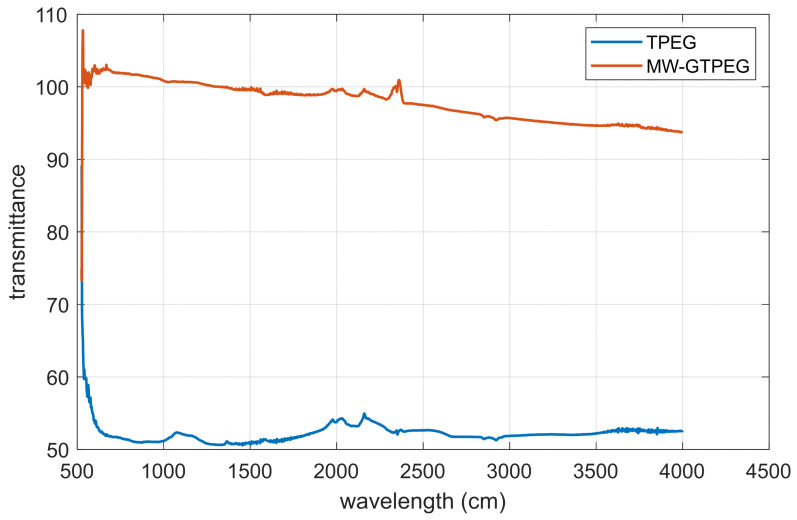
FTIR analysis of one scan of MW-GTPEG and one scan of TPEG. The wavelength resolution is 0.482 cm.

Figure 2 shows the FTIR spectra corresponding to TPEG and MW-GTPEG. In general, the two substances exhibited similarities, while TPEG had a higher level of absorption in comparison to MW-GTPEG. The observed spectra exhibit congruence with the findings of a prior investigation undertaken by Cuccarese et al. (2023) [43]. The researchers reached the conclusion that there was no discernible peak specifically associated with functional groups. Additionally, they verified that the structure of TPEG is comparable to that of pure graphitic substances, as previously reported [21]. Furthermore, the thermo-plasma expansion of TPEG and MW irradiation of MW-TPEG were performed inertly in this study, which ensured no oxidation of the graphite. Our results demonstrated that the procedures we performed on the TPEG were successful in producing a MW-GTPEG with indiscernible differences.

More specifically, commercial TPEG showed weak and poorly resolved absorption bands, consistent with its predominantly graphitic structure and the limited amount of infrared-active functional groups. After alginate-based granulation and microwave treatment, MW-GTPEG showed weak spectral contributions in regions typically associated with alginate-based materials. In particular, a weak relative minimum around 1600–1620 cm^−1^ may be attributed to the asymmetric stretching vibration of carboxylate groups (−COO−) from the alginate matrix, possibly overlapped with C=C vibrations of graphitic domains. A weak contribution in the 1100–1000 cm^−1^ region may be assigned to C–O and C–O–C stretching vibrations of the polysaccharide structure of alginate. No clearly distinguishable broad O–H stretching band was observed in the 3200–3600 cm^−1^ region, suggesting that hydroxyl-related contributions were weak, poorly resolved, or partly reduced after microwave treatment. Overall, the FTIR results support the presence of the alginate-based matrix in MW-GTPEG, while no clear evidence of extensive oxidation or chemical functionalization of the graphitic phase after microwave treatment was observed.

#### 3.1.2. BET Surface Area and Pore Analysis

Table 2 provides a comparison of physical characteristics between TPEG and MW-GTPEG. The MW-GTPEG material comprises macropores, which make up around 8% of its structure and are commonly referred to as the main pathways for carbon pores. Additionally, micropores and mesopores play a key role in increasing the interior surface area of the material [50]. As anticipated, the granulation process resulted in a decrease in the BET surface area of TPEG from 48 to around 10 m^2^ g^−1^. Additionally, it was observed that the mean pore size of TPEG had a reduction from 117 to 65 Å following the processes of granulation and microwave irradiation. Moreover, the pore size range of the MW-GTPEG material exhibited an increase to 4000 Å, which corresponds to the macropore scale. The findings of the BET analysis align with the corresponding analyses conducted in the study by Cuccarese et al. (2021) [21], again suggesting that we have produced MW-GTPEG successfully.

In general, the formation of pores in MW-GTPEG mostly results from thermal expansion occurring from the evaporation of the CaCl_2_ solution. The observed range of expansion of pore diameter in this investigation matches the findings of Liu et al. (2020), who hypothesized that the MW-induced expansion from the interior to the exterior might plausibly account for the growth of pores in terms of both number and diameter [50].

#### 3.1.3. CHNS, Ash Content, and Point of Zero Charge pH (pHpzc)

The results for the percentage of carbon, hydrogen, nitrogen, and sulfur, along with ash content and pHpzc, are provided in Table 3.

TPEG had more than 90% of carbon as a carbon-based material; in contrast, the percentage of carbon decreased in MW-GTPEG since this composite was prepared using sodium alginate and CaCl_2_. This was also apparent in the higher ash content of MW-GTPEG. Moreover, the percentages of nitrogen and sulfur could be considered almost negligible in both materials, while the hydrogen content increased in MW-GTPEG. The pHpzc of TPEG increased from 3.0 to 5.3 after MW irradiation. Having the solution pH value higher than the pHpzc, where the surface of the adsorbent will be negatively charged, will facilitate the adsorption of cationic dyes such as MB.

### 3.2. Optimization of Adsorption Process Parameters Using RSM

RSM is a helpful analytical and statistical method for assessing various independent parameters’ effects on the response. Different trials were conducted for several initial parameters, applying statistically sketched tests to explore the combined impact of the factors (Table S1). RSM provides data on the linear influence of the parameters on the adsorption response, as well as a view of the parameters’ squared and interaction influences. Table S2 shows the analysis of variance (ANOVA) for three responses (optimized results) from RSM; values less than 0.05 indicate significant models.

Moreover, Table 4 presents the coefficients determining R^2^ for reduced quadratic responses. The R^2^, predicted R^2^, and the adjusted R^2^ values are all very high. The R^2^ value shows how the model explains the data, however, a higher number of data points may skew the R^2^ value to a higher value. The predicted R^2^ value determines how well a model can predict the data, and the adjusted R^2^ considers the number of parameters in the model, thus a higher adjusted R^2^ means a new added parameter improves the model fit more than by chance. In this case, a high R^2^, predicted R^2^, and adjusted R^2^ values mean that the model variables are explaining and predicting the data well given the number of parameters here. The adsorption capacity (q) and adsorption rate were applied to optimize the experimental conditions efficiently since the values of R^2^ were in a satisfactory domain and proved RSM’s accuracy in predicting adsorption behavior and data analysis.

**Figure 3:**
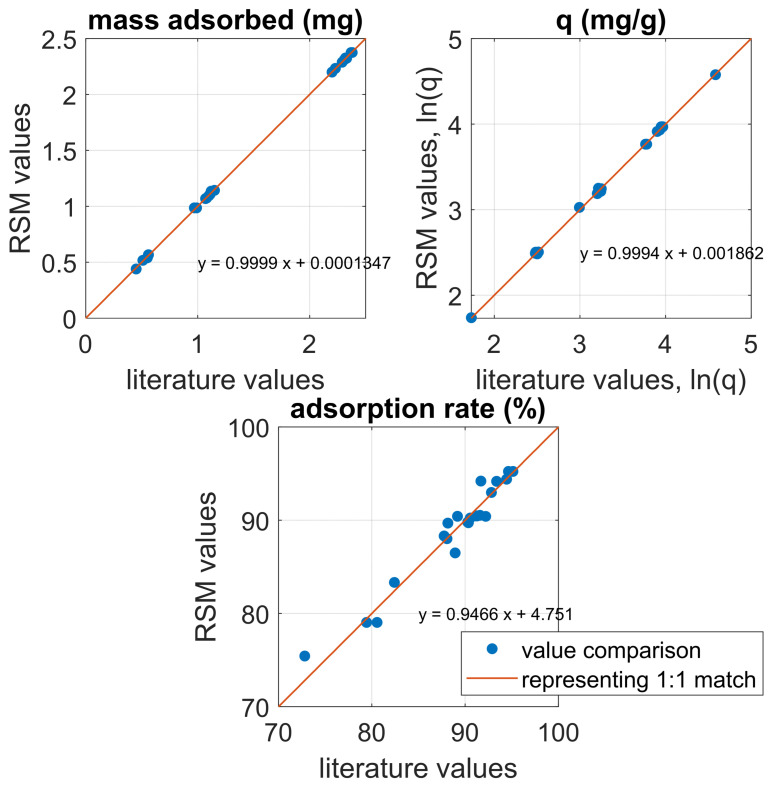
RSM values vs. literature values.

Furthermore, the model’s accuracy was evaluated by plotting literature values versus RSM values in Figure 3, showing the ability of RSM to be used for further optimization.

### 3.3. Evaluation of the One-Factor Effect on Adsorption Features

**Figure 4:**
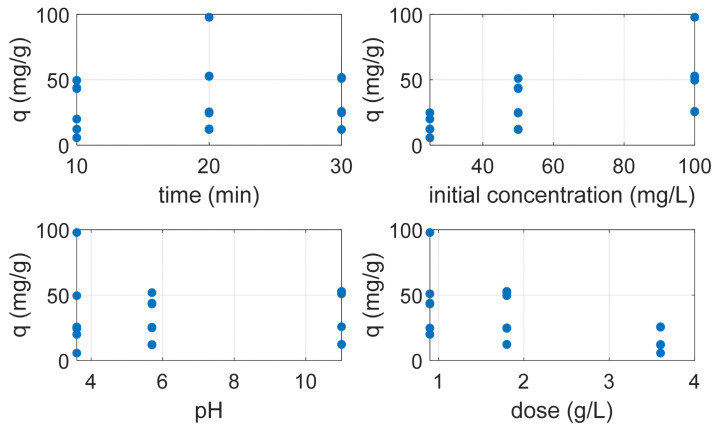
Effects of individual factors on q performance.

**Figure 5:**
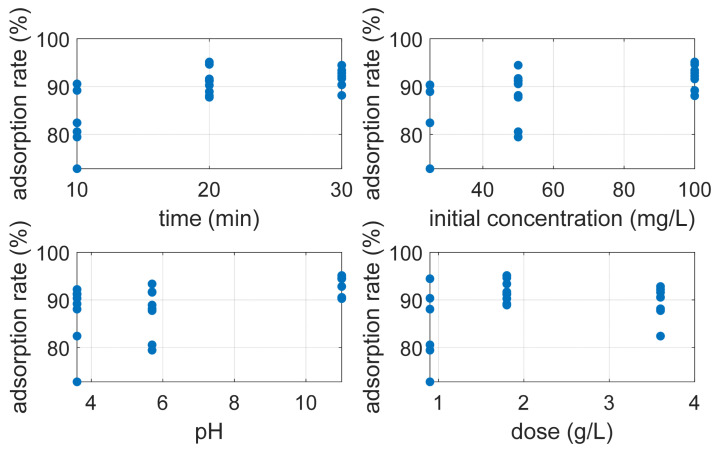
Effects of individual factors on adsorption rate.

The effects of individual factors on q and the adsorption rate are provided in Figure 4 and Figure 5. There was a trend of increasing q and the adsorption rate with increasing the contact time and initial concentration. The reason may be that there was more contact time between MB and the MW-GTPEG pores as well as more adsorbate available with a higher concentration that could be adsorbed in the pore site, respectively. Moreover, both responses were dependent on higher pH values than the pHpzc since, at higher pH values, the surface of the adsorbent is negatively charged, which can pave the way for a cationic dye such as MB to be adsorbed in the pores. Furthermore, the adsorption rate peaked between 1.8 and 2.7 g L^−1^ (Figure 5). In contrast, the downward trend in q (Figure 4) was presumably because it had a dose-response relationship—the higher the dose of adsorbent, the lower the value of adsorption capacity. From this analysis, we decided to investigate the experimental conditions of 20 min of contact time, 100 mg g^−1^ of concentration, pH 5.7, and 0.9 g L^−1^ of dose.

### 3.4. Response Surface Plots

One of the advantages of using RSM is that it provides 3-D plots of the response surface, which clearly illustrates the interactions among the efficient factors and investigates the optimal values of all factors to trigger a maximum adsorption rate and amount.

#### 3.4.1. The Effect on q Due to Contact Time, Initial Concentration, pH, and Dosage

**Figure 6:**
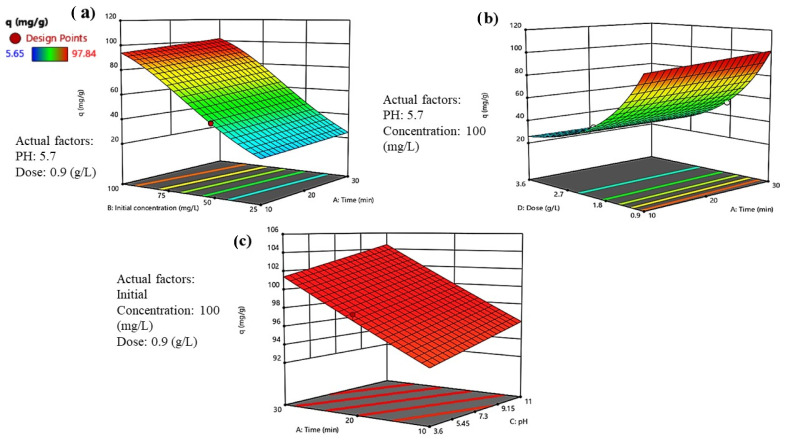
A 3-D surface of the adsorption capacity (q) of MB versus (**a**) initial concentration and contact time, (**b**) dose and contact time, and (**c**) contact time and pH.

**Figure 7:**
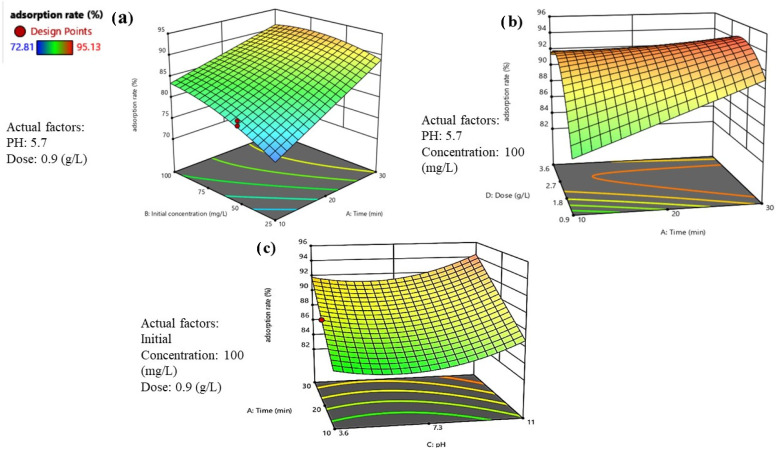
A 3-D surface of the adsorption rate of MB versus (**a**) initial concentration and contact time, (**b**) dose and contact time, and (**c**) contact time and pH.

The MB rate and adsorption capacity (q) versus the contact time, initial concentration, pH, and dosage were plotted on the 3-D surfaces (Figure 6 and Figure 7). As shown in Figure 6b (time-dose interaction), the surface (dose = 0.9 g L^−1^) and minimum time of 10 min along with other fixed factors (pH = 5.7, concentration = 100 mg L^−1^) was predicted to reach the highest adsorption capacity (red colors, 80–100 mg g^−1^), emphasizing that q is highly dependent on the dose of adsorbent. In addition, in all three-time intervals and at the dose of 0.9 g L^−1^, the responses achieved a higher q value, showing that the time factor plays a less dominant role in q changes. The time-initial concentration interaction (Figure 6a) revealed that with an initial concentration greater than 75 mg L^−1^ while other factors (pH 5.7 and dose 0.9 g L^−1^) were held constant for 10 min, q would reach its maximum values (80–100 mg g^−1^), possibly owing to the fact that more MB molecules would have higher probability to be exposed to adsorption sites at higher concentrations. Moreover, increasing both factor time and concentration resulted in the highest values of q, which shows that both factors combined have a direct effect on the adsorption process. This phenomenon may be attributed to the higher presence of the dye at higher concentrations which led to more adsorption, given a longer contact time.

#### 3.4.2. More Adsorption from Higher pH, Longer Contact Time, and Higher MB Concentration

It is worth mentioning that according to Figure 6c, the higher pH resulted in a higher q (104 mg g^−1^), which shows more interaction between the cationic MB and the negative charge of MW-GTPEG pores. Furthermore, the effects of time-initial concentration (Figure 7a) on adsorption rates illustrated that to achieve higher efficiency (>90%), 30 min and more than 35 mg L^−1^ MB solution would be required while keeping other factors constant. Longer time and higher concentration leading to a higher adsorption rate is likely due to having more MB molecules available to be adsorbed for a longer time and subsequently resulting in more mass transfers to the pore structure of the adsorbent [43].

The plot for time-dose interaction (Figure 7b) showed that 10 min of contact time and 1.8 g L^−1^ of MW-GTPEG would favor an adsorption rate of >90%. Also of note, the interaction of higher doses and contact time was predicted to have a direct relationship with the rate of adsorption, likely due to the presence of more adsorption sites in the adsorbent and more mass transfer over a longer period of time. Finally, the pH and time interaction plot (Figure 7c) demonstrated that obtaining >90% efficiency at lower pH required more than 25 min, while for more basic pH, the time was reduced to 18 min.

#### 3.4.3. RSM Results for Optimizing Experimental Conditions

Given that one of the benefits of RSM is keeping one parameter fixed at a time and obtaining the optimal values for other parameters, resulting in the maximum response or target objective, to save materials and time, we modeled for the optimum conditions for achieving the maximum response for both q and the adsorption rate (Table 5). This result was obtained by minimizing the dose and the contact time and keeping the pH of the solution at 5.7, which was close to the real condition and did not need further pH modification. We found that there was more than one solution to obtain maximum responses, and the first solution was the was the best among the conditions tested based on higher desirability (Table S3).

The reliability of the model was also assessed with the RSM verification tool to predict the results of a single experiment, which were then compared to the actual experimental results, demonstrating the model’s predictive accuracy (Table 6).

#### 3.4.4. The Impact Each Variable on MB Removal and Rate

**Figure 8:**
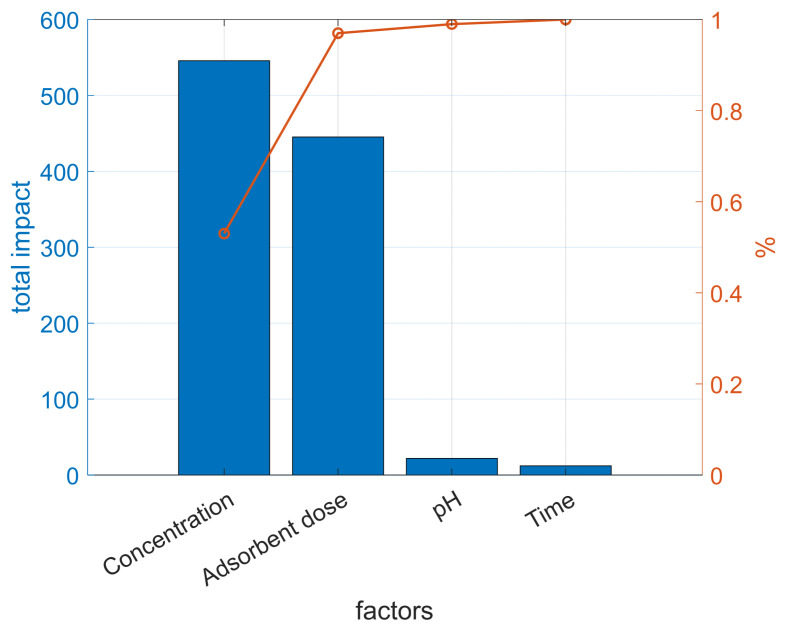
The effects of Pareto design of experiment factors on the adsorption capacity, q.

The impact of driving variables on MB removal was further examined. Pareto charts were used to determine the main factor that influences the adsorbent’s performance based on the analysis of experimental data. Figure 8 shows the Pareto chart for adsorption capacity (q). To create the Pareto chart, the impact of each factor on the response q was calculated, followed by the calculation of the total impact, which means the sum of squared differences between the average response of each level and the overall average response, respectively. Our chart corroborated the results above that initial concentration and adsorbent dose both played a large role in adsorption performance, impacting cumulatively 97% of the adsorption performance; whereas the other two factors (pH and time) had less impact on the adsorption process.

**Figure 9:**
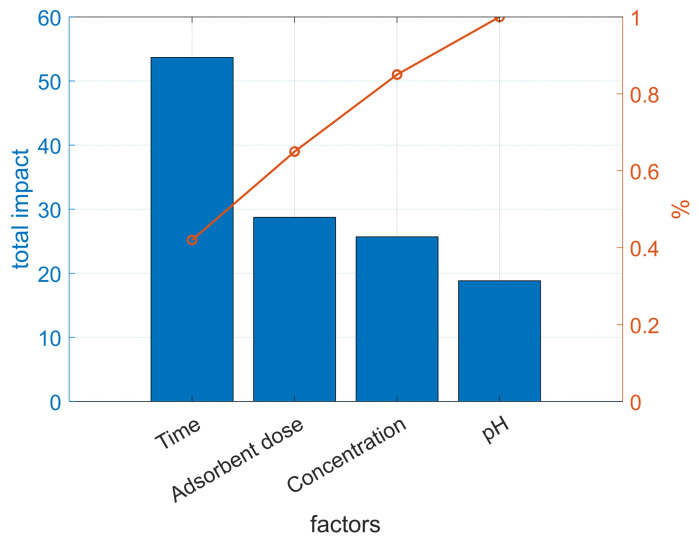
The impact of Pareto design of experiment factors on the adsorption rate.

The impact of variables on adsorption rate is shown in Figure 9. Unlike adsorption capacity, time played a large role in adsorption rate, similar to what our RSM results showed, accompanied by adsorbent dosage (cumulatively 65% of the total impact). Although pH and initial concentration had lower impact on adsorption rates, initial concentration, time, and adsorbent dosage may have >80% of the impact on adsorption rate.

### 3.5. Adsorption Isotherms

**Figure 10:**
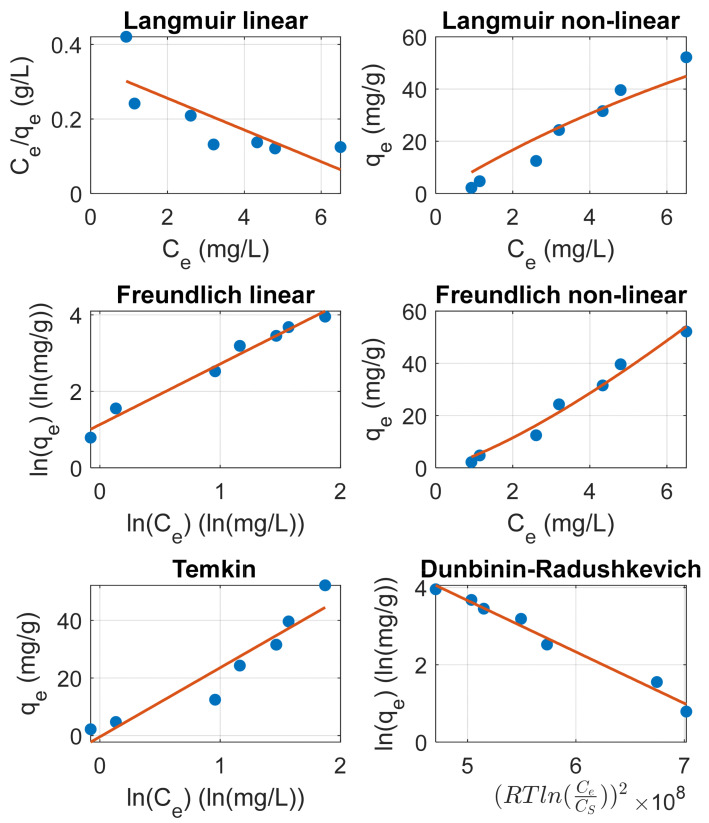
Experimental data fitted to Langmuir, Freundlich, Temkin, and Dubinin-Radushkevich models.

Experimental data were fit to Langmuir, Freundlich, Temkin, and Dubinin-Radushkevich isotherm models are shown in Figure 10. Based on these graphs and the coefficient of determination (R^2^) value, the data were most well-explained by the Freundlich and the Dubinin-Radushkevich models. The parameters of all isothermal models are presented in Table 7. We performed the Chi-squared test for the Freundlich linear and non-linear models and the Dubinin-Radushkevich model to check with the data if there was a significant difference between the models and the observed data. The observed data were found to be not significantly different (*p* > 0.05) from both of the Freundlich models, but significantly different for the Dubinin-Radushkevich model. Therefore, the Freundlich model reflects the heterogeneous surfaces and adsorption consistent with a multiplayer adsorption assumption and the exponential placement of active sites and their related energies, where the adsorbent includes many nearby sites.

### 3.6. Kinetics Studies

**Figure 11:**
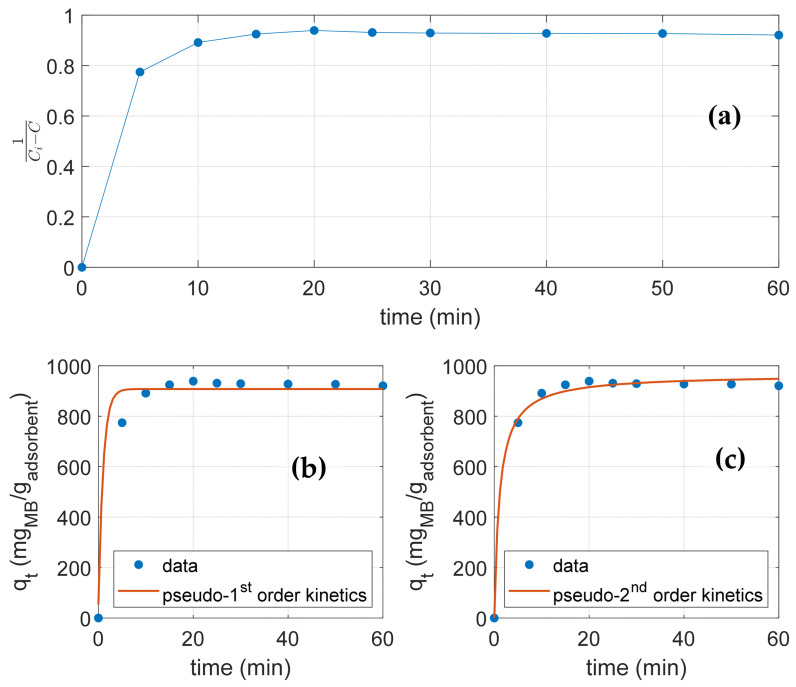
Kinetics (**a**), pseudo-first order and pseudo-second order models ((**b**) and (**c**), respectively).

The study’s kinetics and equilibrium are shown in Figure 11a,b. The pH was set to 5.7, the dose was 1.8 g L^−1^, and the initial concentration was 100 mg L^−1^, with the time intervals of 5 min in the first 30 min and 10 min in the last 30 min. The experiment showed that around 90% of the MB solution was adsorbed in the first 10 min of the test, and equilibrium was achieved at around 94% after 20 min. The kinetics models, namely pseudo-first order and pseudo-second order, were also plotted in Figure 11b,c. The R^2^ values appeared to show that the pseudo-second order kinetics model explained better the kinetics of MB adsorbing onto the MW-GTPEG. To investigate this further, we performed the Chi-squared test on the experimental data against both kinetics models. We found that only the pseudo-second order kinetics model was not significantly different (*p* > 0.05) from the experimental data. This shows that the adsorption of MB onto MW-GTPEG followed more closely with the second order kinetics model. Table 8 shows the kinetics parameters for both models.

### 3.7. Thermodynamics Output

**Figure 12:**
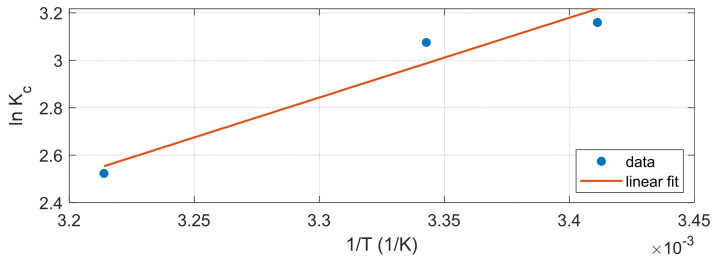
Thermodynamics plot of the adsorption process.

Figure 12 and Table 9 show the thermodynamics of the adsorption process. The negative values of Gibbs free energy (ΔG^0^) illustrated that the adsorption process is thermodynamically favorable, feasible, and spontaneous. In addition, the positive value of enthalpy (ΔH^0^) shows that the MB adsorption on MW-GTPEG was endothermic. However, we would like to caution that our thermodynamic results were from limited experimental conditions, therefore the values may change over a larger range of temperatures.

Potential mechanisms and pathways that can facilitate physical adsorption include electrostatic attraction, π-π interactions, van der Waals forces, hydrogen bonding, acid-base reactions, hydrophobic interactions, and ion exchange. The primary method of MB removal in this research might have been dominated by physical adsorption, whereby the mechanism of adsorption was most likely the weak van der Waals forces; however, confirming van der Waals forces requires an experimental setup that tests for increased adsorption with increase specific surface area [51], which was not part of our experimental goals. It is important to note that physical adsorption is a reversible phenomenon. Although pore-filling interaction could also be possible, which requires confirmation through the curve of adsorption potential density versus adsorption volume, without examining the curve of adsorption potential density versus adsorption volume [52], our experimental setup was not suitable to confirm it. In addition, the BET data (Table 2) revealed that the presence of mesopores and macropores in MW-GTPEG possibly aided the process of physical adsorption by increasing the surface area.

#### Comparison of MW-GTPEG, GTPEG, and TPEG Powders in Terms of Adsorption Performance

**Figure 13:**
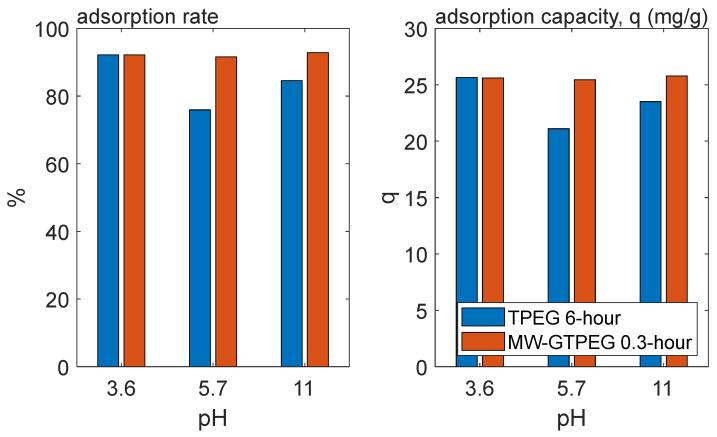
Comparison between TPEG powder and MW-GTPEG adsorption performance in different pH ranges.

Experiments were conducted to examine the performance of different graphite adsorbents utilized in this study. Figure 13 shows the differences between TPEG powders and MW-GTPEG, where the adsorption time for TPEG powders was 6 h and 0.3 h for MW-GTPEG. In general, MW-GTPEG showed superiority over TPEG powders in adsorption rates and capacity. It may be attributed to the different characteristics of the two adsorbents: since TPEG powders are hydrophobic, it took longer to adsorb the MB molecules, whereas MW-GTPEG showed hydrophilic interaction with the MB dye molecules.

**Figure 14:**
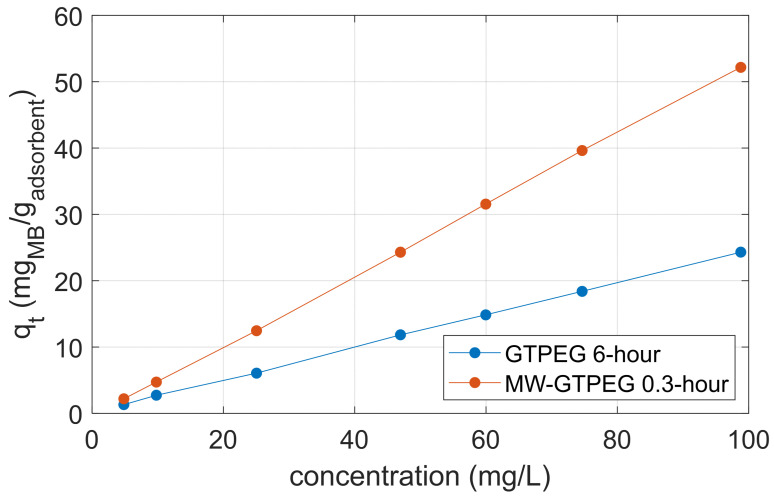
Equilibrium of MB removal by different adsorbents (GTPEG and MW-GTPEG).

**Figure 15:**
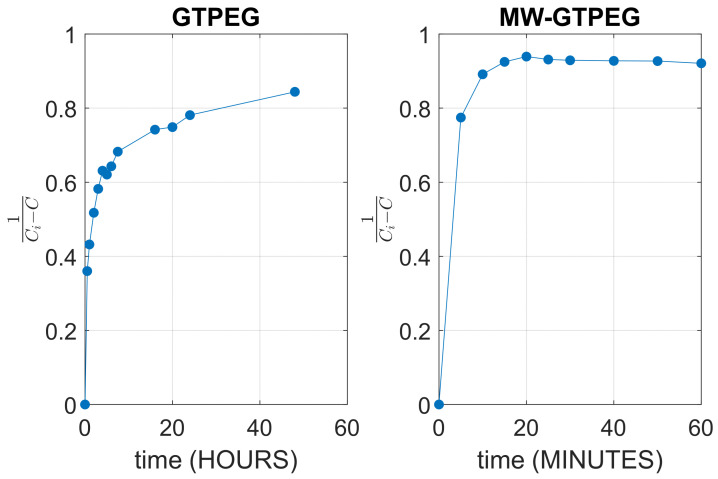
Time to reach equilibrium in MB removal by different adsorbents (GTPEG and MW-GTPEG).

Figure 14 and Figure 15 show the differences in equilibrium and in adsorption time to reach equilibrium between MW-GTPEG and GTPEG. It should be noted that the contact time of GTPEG was 6 h versus 0.3 h for MW-GTPEG. The graphs revealed that the performance of these materials was closer at lower concentrations; by increasing concentrations of MB, MW-GTPEG showed almost 2.5 times higher adsorption capacity (in 100 mg L^−1^ around 20 and 50 mg g^−1^ for GTPEG and MW-GTPEG, respectively), which may be related to the physical characteristics of the two adsorbents, since MW would have created a cleaner pore structure for the MW-GTPEG adsorbent [43]. The adsorption time to reach equilibrium of these studies demonstrated a higher rate from MW-GTPEG by reaching equilibrium in 20 min, while it took around 24 h for GTPEG.

A review of recent literature on modified EG, its adsorption performance, and their adsorption capacities is provided in Table 10 as a comparison with this study.

As a preliminary test to check the adsorption of MW-GTPEG of MB from natural surface water in the presence of natural organic and inorganic matter, two preliminary experiments were conducted for potential competition for adsorption sites (details in Text S1). The preliminary results show that about 96% MB was removed. This suggests the viability of using MW-GTPEG to remove contaminants such as MB from environmental waters. Future studies are needed to investigate the application of MW-GTPEG further.

### 3.8. Regeneration Studies

**Figure 16:**
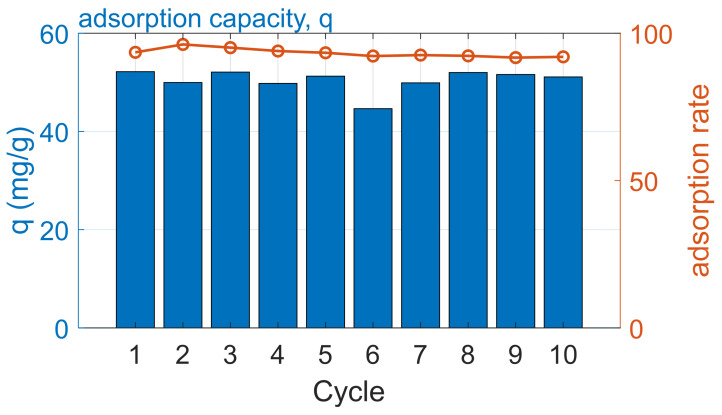
Regeneration of MW-GTPEG by MW irradiation.

A commercial MW system was used to conduct the thermal regeneration of MW-GTPEG exhausted by MB. A medium power level of p6 (1100 ± 20 watts) and 5 min of irradiation time were sufficient to remove MB from adsorbent pores and prepare them for the next cycle. In terms of energy usage, each regeneration cycle used 0.08 ± 0.01 kWh. The results of 10 cycles of regeneration of MW-GTPEG (Figure 16) showed no decrease in adsorption capacity or rates, emphasizing MW irradiation as a promising tool for sustainable recycling. Aside from the easy process of MW regeneration, owing to the lower energy consumption, MW can offer an environmentally friendly regeneration process compared to conventional thermal regeneration. After 10 cycles of regeneration, the weight loss of MW-GTPEG was around 8%, while conventional regeneration processes (ex., thermal regeneration, etc.) do not cause such a high material loss to the same extent at the end of cyclic regeneration [53].

In the field of environmental technology, cutting-edge green technology that uses less energy, produces less waste, and reuses materials is essential for a more sustainable and competitive product. Microwave oil extraction has been found to be superior to traditional methods in terms of product purity and processing time, thus representing a potential area for future study and development. Various studies have highlighted the efficacy of MW technology in the extraction of oil from various materials [54]. Kusuma et al. (2017) found that compared to conventional oil extraction methods, MW only needs to be used 4.6113 times less often, leading to the conclusion that this method is more energy-efficient overall [54]. The new MW technique resulted in less relative CO_2_ emissions (kg g^−1^ of oil) compared to the traditional approach (4.61 times more) [54,55,56,57]. In this study, 0.02 kWh of energy was needed to dry or purify GTPEG that had been soaked in DDW using MW, whereas the same task in a normal oven would have required 0.2 kWh of energy. Furthermore, we calculated that regeneration only required 0.08 kWh of energy using MW, whereas it required much more energy using a small oven (>1 kWh) since most of the energy was spent on warming the oven chamber to a specific temperature. Since CO_2_ emissions are proportional to energy consumption, for material drying and regeneration in our study, MW generally released much less CO_2_ per g of absorbent compared to conventional methods [58,59].

## 4. Conclusions

A microwave granular thermo-plasma-expanded graphite (MW-GTPEG) was prepared from thermo-plasma-expanded graphite (TPEG) to remove the cationic dye, methylene blue (MB), from aqueous solutions via adsorption. A commercial microwave (MW) system was also employed to prepare the MW-GTPEG for regeneration. In addition, the response surface methodology (RSM) was used to optimize multiple important parameters that may influence the efficiency of adsorption. Using the results from RSM as a guide, the adsorption experiments showed that the MW-GTPEG was an efficient adsorbent for removing the MB from deionized distilled water (DDW) and from a natural surface water sample. Moreover, applying RSM facilitated the workflow of this study by decreasing the number of runs and consequently triggering the saving of materials and time. Furthermore, our calculation showed that MW irradiation was an economical option for regeneration studies because of its low energy consumption, its ability to regenerate adsorbent without the loss of q, and the fact that this method of regeneration resulted in a low weight loss of the adsorbent after regeneration. The results of this study showed the following:

- BET surface analysis for both TPEG and MW-GTPEG showed that the surface area decreased for the granular form (MW-GTPEG) compared to TPEG, while a macropore structure was observed for MW-GTPEG [43], highlighting the importance of this analysis;
- The Pareto chart revealed that initial concentration and adsorbent dose were the main factors affecting q in our adsorption study, and the regeneration studies showed that MW irradiation could work for 10 consecutive cycles by offering less energy consumption (0.08 kWh), a faster process (less than 5 min), and less material losses (around 8%) in comparison to conventional regeneration;
- Preliminary data showing the removal of MB by MW-GTPEG from a real sample of lake water demonstrated its potential application in real polluted freshwater media that contain natural organic matter and inorganic compounds;
- Comparison studies on TPEG, GTPEG, and MW-GTPEG highlighted the shorter time for MW-GTPEG and its higher q and adsorption rate than those of GTPEG and TPEG powder;
- Energy consumption of regenerating adsorbent with MW is substantially lower than that of conventional methods; similarly, CO_2_ emissions are also much lower.

This study demonstrated the potential of using MW-GTPEG to remove contaminants from water in an energy-efficient way. From the results of this study, future studies can be designed to investigate the application of MW-GTPEG more in-depth with different environmental waters containing different contaminants.

## Figures and Tables

**Table 1:** The domain of parameters optimized in the RSM method [47].

Factor	Name	Units	Number of Levels	Values
A	Time	min	3	10–20–30
B	Initial concentration of MB	mg L^−1^	3	25–50–100
C	pH	-	3	3.6–5.7–11
D	Dose	g L^−1^	3	0.9–1.8–3.6

**Table 2:** Physical characterization of adsorbents (details in Figure S1).

Materials	BET (m^2^ g^−1^)	Total Pore Volume (cm^3^ g^−1^)	Average Pore Diameter (Å)	Density Functional Theory (DFT) Pore Size Range (Å)	Porosity Distribution by N2–DFT Model	
					Mesopore (cm^3^ g^−1^)	Macropore (cm^3^ g^−1^)
TPEG	48	0.1329	117	8.5–343	0.1329 (100%)	0 (0%)
MW-GTPEG	10	0.0195	65	14.8–4003	0.018 (92%)	0.0014 (8%)

**Table 3:** Chemical characteristics of TPEG and MW-GTPEG [47].

Materials	Carbon %	Hydrogen %	Nitrogen %	Sulfur %	Ash Contents %	pHpzc
TPEG	91.59	0.26	0.05	0.91	1.8	3.0
MW-GTPEG	32.35	4.38	0	0	12.3	5.3

**Table 4:** Optimization statistics [47].

Response	Adsorption Capacity (q) Reduced Quadratic Model	Adsorption Rate (%) Reduced Model
R^2^	0.9994	0.9466
Adjusted R^2^	0.9991	0.9097
Predicted R^2^	0.9983	0.8134

**Table 5:** Results of optimization of RSM.

Number	Time (min)	Initial Concentration (mg L^−1^)	pH	Dose (g L^−1^)	q (mg g^−1^)	Adsorption Rate (%)	Desirability
1	10	100	5.7	0.9	93.95	83.5	0.829
2	10	100	5.7	1.8	50.54	89.7	0.789
3	20	100	5.7	0.9	98.03	87.3	0.755
4	20	100	5.7	1.8	51.73	91.9	0.686
5	20	50	5.7	0.9	46.37	85.1	0.671

**Table 6:** Verification of RSM model.

Analysis	Predicted Mean	Predicted Median	Std Dev	95% Prediction Interval (PI) Low	Observed	95% Prediction Interval (PI) High
q (mg g^−1^)	51.74	51.73	1.04	49.34	51.40	54.23
adsorption rate (%)	91.93	91.93	1.63	87.83	96.0	96.02

**Table 7:** Isotherm parameters from different adsorption models.

Model	Parameters		
Langmuir	K_L_	q_max_	R^2^
Langmuir linear	−0.1249	−23.49	0.6223
Langmuir non-linear	0.05108	180.1	0.8988
Freundlich	n_f_	K_f_	R^2^
Freundlich linear	0.6307	3.096	0.9783
Freundlich non-linear	0.7607	4.617	0.9794
Temkin	B_1_ = 23.98	A = 0.9833	R^2^ = 0.8984
Dubinin-Radushkevich	E = 8668	q_s_ = 30,300	R^2^ = 0.9811

**Table 8:** Kinetics parameters for pseudo-first and pseudo-second order models.

Model	q_e_ (mg g^−1^)	Rate Constant	R^2^
pseudo-first order	908.1	k_1_ = −0.00095	0.9742
pseudo-second order	965.9	k_2_ = −0.00093	0.9957

**Table 9:** Thermodynamics parameters.

T (K)	Kc	ΔG^0^	ΔH^0^	ΔS^0^	R^2^
293	23.56	−7700	2403 × 10^6^	8,197,000	0.9497
299	21.65	−7648			
311	12.46	−6526			

**Table 10:** Results of recent studies on modified EG and the result of this study.

References	Materials	Adsorption Performance	Time	Other Conditions
[28]	Modified EG powder	Langmuir q_max_ = 7.77 μg g^−1^ (q_exp_ = 2.7 μg g^−1^; recalculated here to be 2700 mg g^−1^)	180 min	pH: 7 dose: 3.5 g L^−1^
[1]	Microwave-assisted EG	Langmuir q_max_ = 47.5 mg g^−1^ (q_exp_ = 51 mg g^−1^)	80 min	pH: 9 dose: 2 g L^−1^
[49]	Graphene oxide calcium alginate	Langmuir q_max_ = 181 mg g^−1^	300 min	pH: 5.4 dose: 0.5 g L^−1^
[30]	Magnetic EG Fe_3_O_4_/3MEG-I	(q_exp_ = 3 mg g^−1^)	60 min	pH: 7.09 -
[29]	Modified expanded graphite/Fe_3_O_4_	Langmuir q_max_ = 79 mg g^−1^	140 min	- dose: 3.3 g L^−1^
This study	MW-GTPEG	Langmuir q_max_ = 180.13 mg g^−1^ (q_exp_ = 97.84 mg g^−1^)	20 min	pH: 5.7 dose: 0.9 g L^−1^

## Data Availability

The original contributions presented in this study are included in the article/Supplementary Material. Further inquiries can be directed to the corresponding author.

## Supplementary Materials

The following supporting information can be downloaded at: https://www.mdpi.com/article/10.3390/ma19132884/s1, Text S1: The performance of adsorption of MB from surface water; Text S2: Information regarding selecting MW irradiation power and duration; Table S1: Different runs of MB removal; Table S2: Analysis of variance for three responses (optimized results) from RSM in terms of *p*-values; Table S3: Results of optimization of RSM; Table S4: Advantages and disadvantages of different dye treatment methods; Table S5: Equations and parameters for isothermal models; Table S6: Kinetics models used in this study starting with the generic rate equation d(q_e_ − q_t_) = −k_n_ (q_e_ − q_t_)^n^; Table S7: Thermodynamic equations of adsorption; Table S8: Details of the commercial microwave oven; Figure S1: Linear isotherm plots and pore width versus dV/dlog(W) pore volume of adsorbents. References [48,60,61,62,63,64,65,66,67,68] are cited in the Supplementary Materials.

## Abbreviations

The following abbreviations are used in this manuscript:

AC	Activated carbon
AOP	Advanced oxidation process
BET	Brunauer–Emmett–Teller
BOD	Biochemical oxygen demand
COD	Chemical oxygen demand
DDW	Deionized distilled water
EG	Expanded graphite
EPA	Environmental Protection Agency
FD&C	Food, drugs, and cosmetic colorant
FTIR	Fourier transform infrared spectroscopy
GIC	Graphite intercalation compound
GTPEG	Granular thermo-plasma expanded graphite
MB	Methylene blue
MEG	Modified expanded graphite
MW	Microwave
MW-GTPEG	Microwave-granular thermo-plasma expanded graphite
PFAS	Per- and polyfluoroalkyl substances
RCRA	Resources Conservation and Recovery Act
RSM	Response surface methodology
TPEG	Thermo-plasma expanded graphite
