# Supplementary material for: Microwave-Assisted Production and Regeneration of Granular Expanded Graphite for the Adsorption of Cationic Dye Methylene Blue from Aqueous Solutions
Source: Materials (Basel). 2026 Jul 6;19(13):2884. doi: 10.3390/ma19132884 (PMC13363074; doi:10.3390/ma19132884)
Supplement: Supplementary file 1 [file materials-19-02884-s001.zip › materials-4346904-supplementary.pdf]

Supplementary Information

Microwave-Assisted Production and Regeneration of Granular Expanded Graphite for the Adsorption of Cationic Dye Methylene Blue from Aqueous Solutions

A. Faraji [1, 2*] , D. Caniani [3] , D. Bersano [4] , S. Masi [1] , I.M. Mancini [3] , L.Y. Tseng [5], T. Karanfil [2]

[1] Department of Engineering, University of Basilicata, viale dell'Ateneo Lucano, 10, 85100 Potenza, Italy
[2] Department of Environmental Engineering and Earth Sciences, Clemson University, 230 Kappa Street, Clemson, SC 29634, USA
[3] Department of Health Sciences, University of Basilicata, viale dell'Ateneo Lucano, 10, 85100 Potenza, Italy
[4] Eni S.p.A. - Natural Resources, Wind and Marine Energy Research Center, Via Felice Maritano, 26 - 20097 San Donato Milanese (MI) – Italy DB: delfina.bersano@eni.com
[5] Civil Engineering Department, The City College of New York, 160 Convent Avenue, New York, NY 10031, USA

*Corresponding author, afaraji@g.clemson.edu

Table of Contents:

Text S1: The performance of adsorption of MB from surface water

Text S2: Information regarding selecting MW irradiation power and duration

Table S1: Different runs of MB removal

Table S2: Analysis of variance for three responses (optimized results) from RSM in terms of p-values

Table S3: Results of optimization of RSM

Table S4: Advantages and disadvantages of different dye treatment methods

Table S5: Equations and parameters for isothermal models

Table S6: Kinetics models used in this study starting with the generic rate equation $d(q_e - q_t) = -k_n (q_e - q_t)^n$

Figure S1: Linear iso-therm plots and pore width versus dV/dlog(W) pore volume of adsorbents

Text S1. The Performance of Adsorption of MB from Surface Water

To test whether MW-GTPEG could be used to remove MB from a natural surface water sample from Lake Hartwell in South Carolina, USA, MB was added to the sample to have a final concentration of 100 mg L-1. Then 1.8 g L-1 of MW-GTPEG was added without pH modification (5.7), and the solution was stirred for 20 min. This sample of surface water had a TOC of 2.00 mg L-1, 2.7 mg L-1 of chloride, 11.3 mg L-1 of phosphate, and an alkalinity of 17 mg L-1. The results showed that MW-GTPEG did not decrease its adsorption of MB in the presence of natural organic and inorganic matter, indicating that MW-GTPEG can adsorb MB in polluted freshwater media with similar chemical characteristics as our natural water sample such as rivers and surface waters. The results of the duplicate experiment for surface water produced a q value of 51.4 mg g-1, corresponding to a 96% removal efficiency. No additional data available for this experiment.

Text S2. Information Regarding Selecting MW Irradiation Power and Duration

We performed preliminary experiments to determine the power and the duration of MW irradiation. Our objective was to remove the water and recover the sample dry mass. Through trial and error, we determined that heating durations longer than 2 min resulted in materials loss, potentially due to the degradation of sodium alginate via heating. We selected 510 W because it removed water effectively without damaging the material while keeping the duration of the irradiation to 2 min. We also experimented with lower power settings, but they required longer heating periods (longer than 4 min). No additional data available for this preliminary experiment.

Table S1. Different runs of MB removal.

	Factor 1	Factor 2	Factor 3	Factor 4	Response 2	Response 3
**Run**	**A: Time**	**B: Initial concentration**	**C: pH**	**D: Dose**	**q**	**adsorption rate**
**Unit**	**min**	**mg L^{-1}**	**pH**	**g L^{-1}**	**mg g^{-1}**	**%**
1	10	50	11	3.6	12.22	90.55
2	30	25	3.6	0.9	24.76	90.35
3	30	50	11	0.9	50.97	94.45
4	10	25	3.6	3.6	5.65	82.42
5	10	50	5.7	0.9	43.2	79.44
6	20	100	11	1.8	52.58	94.64
7	10	100	3.6	1.8	49.54	89.18
8	20	50	3.6	1.8	24.63	91.28
9	20	25	11	1.8	12.37	90.28
10	30	100	5.7	1.8	51.87	93.37
11	20	25	5.7	1.8	12.19	88.93
12	20	50	3.6	1.8	24.6	91.19
13	30	100	3.6	3.6	25.61	92.21
14	20	100	3.6	0.9	97.84	88.06
15	10	50	5.7	0.9	43.8	80.56
16	30	100	11	3.6	25.78	92.82
17	30	50	5.7	3.6	11.98	88.15
18	20	50	3.6	1.8	24.61	91.23
19	20	100	5.7	3.6	25.44	91.6
20	20	50	5.7	3.6	11.93	87.75
21	10	25	3.6	0.9	19.96	72.81
22	20	100	11	1.8	52.85	95.13
23	30	50	5.7	1.8	24.93	91.69

Table S2. Analysis of variance for three responses (optimized results) from RSM in terms of p-value.

Adsorption capacity q (mg g^{-1})	p-value	Adsorption rate (%)	p-value
Model	< 0.0001	Model	< 0.0001
A-Time	< 0.0001	A-Time	< 0.0001
B-Initial concentration	< 0.0001	B-Initial concentration	0.0002
C-pH	0.0148	C-pH	0.0136
D-Dose	< 0.0001	D-Dose	0.0060
AB	0.0250	AB	0.0336
AD	0.0012	AD	0.0018
B^2	< 0.0001	B^2	0.0961
D^2	0.0961	C^2	.0854
Lack of fit	0.0052	D^2	0.0003
		Lack of fit	0.0055

Table S3. Results of optimization of RSM.

Number	Time (min)	Initial concentration (mg L^{-1})	pH	Dose (g L^{-1})	q (mg g^{-1})	Adsorption rate (%)	Desirability
1	10	100	5.7	0.9	93.95	83.5	0.829
2	10	100	5.7	1.8	50.54	89.7	0.789
3	20	100	5.7	0.9	98.03	87.3	0.755
4	20	100	5.7	1.8	51.73	91.9	0.686
5	20	50	5.7	0.9	46.37	85.1	0.671

Table S4. Advantages and disadvantages of different dye treatment methods.

Treatment	Definition	Advantages	Disadvantages	References
Algae degradation	Using algae to consume dyes	Low cost, no harm to the environment	Inconsistent performance, long growth time for fungus, needs large reactors	[60, 61]
Fungus	Self-growing fungus sustained on dye molecules	Simultaneous removal of different dyes		
Advanced oxidation process (AOP)	Generation of oxidizing radicals that break down dye pollutants	Degrade many contaminants	High cost/energy, the production of hazardous by-products, pH dependent	[62]
Ozonation	Ozone generation from oxygen	Effectively removing dyes, there is no sludge generation, rapid reaction	Short half-life time, high cost	[60]
Coagulation	Destabilization of charged suspended and colloidal contaminants	No potentially dangerous intermediates are produced	The generation and subsequent removal of sludge.	[63]
Adsorption	Solid particles migrate from the liquid phase to the surface of the adsorbent material	The capacity to recycle the adsorbents, low cost, high efficiency, suitable for the treatment of persistent dyes	Some adsorbents are costly and need pre-processing and activation	[64, 65]

Table S5. Equations and parameters for isothermal models.

Models	Equations	Parameters	Details
Langmuir	$\frac{C_e}{q_e} = \frac{1}{q_m K_L} + \frac{C_e}{q_m}$ (3)	C_e = concentration of adsorbate at equilibrium (mg L^{-1})<br>q_m = maximum adsorption capacity (mg g^{-1})<br>q_e = adsorption capacity at equilibrium (mg g^{-1})<br>K_L = Langmuir constant related to the free energy of the process (L mg^{-1})	• Adsorption onto an adsorbent surface uniformly with a fixed number of sites [66].<br>• A monolayer of adsorbate reaches equilibrium at saturation on the adsorbent surface.<br>• Assumes the surface coverage with an equilibrium between the relative adsorption and desorption.<br>• The adsorption process is related to the number of available sites on the adsorbent surface, while desorption corresponds to the covered sites in the adsorbent.
Freundlich	$\ln q_e = \ln K_f + \frac{1}{n_f} \ln C_e$ (4)	K_f = a representative of adsorption capacity [(mg g^{-1}) (L mg^{-1})$^{1/n}$]<br>n_f = Freundlich constant equal to adsorption intensity	• Freundlich (1906) suggested an empirical fitting model with heterogeneous surfaces [67].<br>• Adsorption is in a multiplayer manner with an exponential placement of multiple active sites according to their related energies.
Temkin	$q_e = B_1 \ln A + B_1 \ln C_e$ (5)	B_1 = heat of adsorption (J mol^{-1})<br>A = equilibrium binding constant (L g^{-1})	• Assumes an indirect adsorbate/adsorbate interaction.<br>• The increased surface coverage is due to linear reductions of the adsorption process.
Dubinin-Radushkevich [48]	$\ln q_e = \ln q_s - \left(\frac{RT ln\left(\frac{C_e}{C_S}\right)}{E}\right)^2$ (6)	q_s = adsorption capacity (mg g^{-1})<br>C_S = solubility of sorbate (mg L^{-1}); 43600 mg L^{-1}<br>R = ideal gas constant (8.314 J (mol K)$^{-1}$)<br>T = temperature (Kelvin)<br>E = characteristic free energy of adsorption (kJ mol^{-1})	• Provides information regarding the adsorption on the adsorbent sites in terms of physical or chemical interactions.<br>• Assumes the adsorption process in heterogeneous surfaces with a steric hindrance between the adsorbed and nearby adsorbate.<br>• Contemplates a multilayer adsorption by van der Waals forces.<br>• Based on Gaussian energy distribution onto heterogeneous surfaces.

Table S6. Kinetics models used in this study, starting with the generic rate equation $d(q_e - q_t) = -k_n (q_e - q_t)^n$.

Kinetics model	Equation		Parameters
Pseudo-first order	$\ln(q_e - q_t) = -k_1 t + \ln q_e$ $q_t = q_e - \frac{q_e}{e^k}\frac{1}{e^t}$	(7)	q_e = adsorption capacity at equilibrium (mg g^{-1}) q_t = adsorption capacity at time t (mg g^{-1}) k_1 = pseudo-first-order rate constant (min^{-1}) t = time elapsed (min)
Pseudo-second order	$\frac{1}{q_e - q_t} = -k_2 t + \frac{1}{q_e}$ $\frac{1}{q_t} = -\frac{1}{k_2 q_e^2}\frac{1}{t} + \frac{1}{q_e}$	(8)	k_2 = pseudo-second-order rate constant (g mg^{-1} min^{-1})

Table S7. Thermodynamic equations of adsorption.

Equation		Parameters	
$\Delta G^0 = -RT \ln K_a$	(9)	ΔG^0 = standard Gibbs free energy (kJ mol^{-1}) K_a = thermodynamic equilibrium constant (unitless) (≈ $K_c = C_{ad} / C_e$ for a dilute solution) [68]	Enthalpy and entropy of the adsorption can be obtained by plotting ln q_e/C_e versus 1/T
$\Delta G^0 = \Delta H^0 - T\Delta S^0$	(10)	C_{ad} = concentration of adsorbed adsorbate (mg L^{-1}) [68]	
$\ln \frac{q_e}{C_e} = -\frac{\Delta H^0}{RT} + \frac{\Delta S^0}{R}$	(11)	C_e = concentration of adsorbate at equilibrium (mg L^{-1}) ΔH^0 = standard enthalpy (kJ mol^{-1}) T = temperature (K) ΔS^0 = standard entropy (kJ mol^{-1})	

Table S8. Details of the commercial microwave oven.

Parameters	Specifications
Power Source	120 V, 60 Hz
Power Consumption	12.3 Amps, 1460 W
Maximum Cooking Power	1250 W
Power level	10 (each with a multiple of 125 W: 125 W to 1250 W)
Operating Frequency	2450 MHz
Outside Dimensions (W x H x D)	555 mm x 304 mm x 493 mm
Oven Cavity Dimensions (W x H x D)	418 mm x 228 mm x 470 mm
Net Weight	Approx. 32.0 lbs (14.3 kg)
Inverter	Cyclonic

Supplementary Information

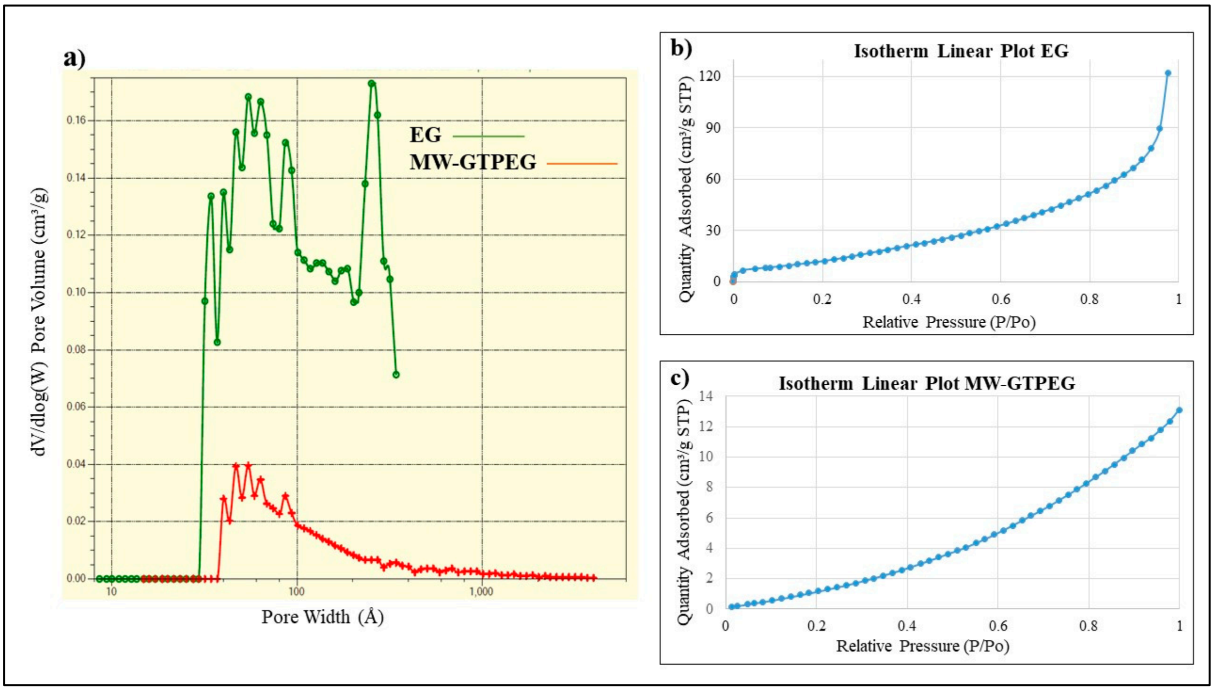

Figure S1. Linear isotherm plots and pore width versus dV/dlog(W) Pore volume of adsorbents.

References

60. Katheresan, V.; Kansedo, J.; Lau, S.Y. Efficiency of various recent wastewater dye removal methods: A review. J. Environ. Chem. Eng. 2018, 6, 4676. https://doi.org/10.1016/j.jece.2018.06.060.

61. Srinivasan, A.; Viraraghavan, T. Decolorization of dye wastewaters by biosorbents: A review. J. Env. Manag. 2010, 91, 1915. https://doi.org/10.1016/j.jenvman.2010.05.003.

62. Gupta, B.; Gupta, A.K.; Tiwary, C.S.; Ghosal, P.S. A multivariate modeling and experimental realization of photocatalytic system of engineered S-C(3)N(4)/ZnO hybrid for ciprofloxacin removal: Influencing factors and degradation pathways. Env. Res. 2021, 196, 110390. https://doi.org/10.1016/j.envres.2020.110390.

63. Sonal, S.; Ugale, D.; Mishra, B.K. Combining Surface Water with Mine Water to Improve the Removal of Natural Organic Matter by Enhanced Coagulation. Mine Water Environ. 2021, 40, 701. https://doi.org/10.1007/s10230-020-00737-z.

64. Manera, C.; Tonello, A.P.; Perondi, D.; Godinho, M. Adsorption of leather dyes on activated carbon from leather shaving wastes: Kinetics, equilibrium and thermodynamics studies. Env. Technol. 2019, 40, 2756. https://doi.org/10.1080/09593330.2018.1452984.

65. Pavithra, K.G.; Jaikumar, V. Removal of colorants from wastewater: A review on sources and treatment strategies. J. Ind. Eng. Chem. 2019, 75, 1. https://doi.org/10.1016/j.jiec.2019.02.011.

66. Langmuir, I. The constitution and fundamental properties of solids and liquids: Part I. JACS. 1917, 39, 1848-1906.

67. Freundlich, H. Kapillarchemie, eine Darstellung der Chemie der Kolloide und verwandter Gebiete, Vol. 1; Akademische Verlagsgesellschaft: Leipzig, Germany, 1930.

68. Liu, Y. Is the Free Energy Change of Adsorption Correctly Calculated? J. Chem. Eng. Data 2009, 54, 1981–1985. https://doi.org/10.1021/je800661q.
